# Physical Fitness, Exercise Self-Efficacy, and Quality of Life in Adulthood: A Systematic Review

**DOI:** 10.3390/ijerph17176343

**Published:** 2020-08-31

**Authors:** María del Rocio Medrano-Ureña, Rosario Ortega-Ruiz, Juan de Dios Benítez-Sillero

**Affiliations:** 1 Faculty of Education Sciences, University of Córdoba, Avenida San Alberto Magno, s/n, 14071 Córdoba, Spain; z62meurm@uco.es; 2 Psychology Department, Faculty of Education Sciences, University of Córdoba, Avenida San Alberto Magno, s/n, 14071 Córdoba, Spain; ortegaruiz@uco.es; 3 Department of Specifics Didactics, Faculty of Education Sciences, University of Córdoba Avenida San Alberto Magno, s/n, 14071 Córdoba, Spain

**Keywords:** physical fitness, exercise self-efficacy, quality of life, adulthood

## Abstract

*Background:* The aim of the present work is the elaboration of a systematic review of existing research on physical fitness, self-efficacy for physical exercise, and quality of life in adulthood. *Method:* Using the Preferred Reporting Items for Systematic Reviews and Meta-Analyses (PRISMA) statement guidelines, and based on the findings in 493 articles, the final sample was composed of 37 articles, which were reviewed to show whether self-efficacy has previously been studied as a mediator in the relationship between physical fitness and quality of life in adulthood. *Results:* The results indicate that little research exists in relation to healthy, populations with the majority being people with pathology. Physical fitness should be considered as a fundamental aspect in determining the functional capacity of the person. Aerobic capacity was the most evaluated and the 6-min walk test was the most used. Only one article shows the joint relationship between the three variables. *Conclusions:* We discuss the need to investigate the mediation of self-efficacy in relation to the value of physical activity on quality of life and well-being in the healthy adult population in adult life.

## 1. Introduction

Today’s developed society is subject to great changes, not always of a positive nature, some of which seem to impact health, well-being, and especially the prolongation of life. Staying healthy is important and has an impact on healthy lifestyle [1]. 

### 1.1. Quality of Life in Adulthood

Adulthood is a period of the life cycle that differs widely due to socio-economic, labor, and cultural conditions. Although it can cover a wide range of ages, current scientific convention specifies an age span that begins between the ages of 40–45 and ends between the ages of 60–65, at which point we can speak of the beginning of old age [2,3]. During the process of adult maturity, important body changes take place or have already taken place, such as menopause and andropause, which involve diverse psychological impacts and, frequently, physiological changes. A loss of bone mass, for example, reduces the strength of the body, making it more vulnerable an injury or disease in daily life [4,5]. People are not always aware of these changes [6,7,8,9,10]. Recently, although there seems to be some interest among the population in understanding the keys to maintaining health and quality of life and to face the decline or deterioration that occurs in old age with better physical and mental health [11], the sedentary life continues to affect a wide range of the adult population [12].

### 1.2. Active Life as a Quality of Life Enhancer

A review study [13] indicated that moderate and systematic physical activity is one of the factors that most affects quality of life. During childhood and adolescence, physical activity is academically programmed, and the habit of physical activity is regulated by schooling, with varying degrees of effectiveness and quality. In old age, health systems and community medicine usually incorporate guidelines that recommend moderate physical activity, with advice on the value of walking, swimming, or going to gyms and social health centers. These efforts, sometimes, are not always successful. However, during the mature adult years [14] that precede old age, the adult population seems to be under pressure from work and family responsibilities, leaving little time for personal attention to preventive health and well-being needs. Some research [15] has revealed the challenge of practicing physical activity or sport in this period of the life cycle. The responsibilities of early adulthood are self-regulated by the experience and years of mature adulthood, and it is at this stage that the practice of physical activity and/or sport for optimal fitness becomes a challenge, because it is known to benefit the individual’s overall health [16,17].

### 1.3. Physical Fitness as an Indicator of Quality of Life

Related to active living and physical exercise is the concept of physical fitness, a well-known and powerful health marker [18,19,20] among middle-aged populations, it is even more powerful than physical activity [7] but we must understand physical fitness as a concept broader than one related exclusively to biological health; it can be defined as the ability to carry out daily tasks with vigor and liveliness, without excessive fatigue, and with enough energy remaining to enjoy leisure time or to cope with unexpected emergencies [21]. Therefore, in addition to being related to biological health, physical fitness is also closely related to psychosocial factors on the human spectrum and has been found to influence fitness parameters [22]. However, few studies present data associating physical fitness in adults with it is psychosocial benefits. It is known that, as a method of achieving general well-being, physical fitness has a large regulated role in the negative relationship between the sedentary life and quality of life [23]. Thus, knowing the levels of physical fitness can be an important tool in providing specific advice to the population in relation to their well-being [24].

However, although it is known that physical activity and improved physical fitness generate benefits and play a fundamental role in both biological and psychological well-being [8,10], it cannot be taken for granted that adults currently incorporate it into their daily routines [8,10]. 

### 1.4. The Role of Self-Efficacy in Maintaining an Active Life

The self-evaluation that is carried out on one’s own activities is called self-efficacy [25]. Expectations of self-efficacy refer to beliefs about personal abilities and the ability to satisfactorily carry out the necessary demands in different situations [26]. Losses inherent to the aging process, such as those related to physical functioning, can affect how one believes in one’s control, or loss of control of self-efficacy [2]. Fortunately, the practice of physical exercise can alleviate these consequences [19]. However, even though people understand the beneficial effects of healthy habits on their own bodies and on their overall well-being and health, we are not sure if there is reciprocity between this knowledge and the integration of physical exercise into their life routines [27].This may seem a paradox in relation to classical theories of motivation towards physical exercise, which emphasize the role of rationality in the decision-making process [28]. It is here that the concept of self-efficacy for physical exercise becomes important, since it determines in part one’s motivation to practice physical activity and is one of its most powerful predictors [29]. 

### 1.5. The Present Study

As a result of these considerations, empirical evidence suggests the important role that the relationship between self-efficacy and the practice of physical activity and exercise performance can play; however, the relationship and influence between self-efficacy and quality of life in terms of physical fitness during mid-life remains relatively limited and therefore does not provide clarifying results. Furthermore, this relationship appears to be very important if we consider that physical fitness is a factor intimately related to well-being and quality of life, as well as a quantitative aspect of each person’s physical functioning—functioning that declines as one ages, therefore, analysis of the relationship between these constructs appears to be an interesting hypothesis for a systematic review. To this end, the general objective of this study was to carry out an exhaustive review of the existing literature delve deeper into this topic. In particular, a specific objective that was established, review the measurement instruments for the specific variables.

## 2. Material and Methods

We selected articles in the PubMed, Scopus, Web of Science, PsycINFO, database presenting research results on the relationship between quality of life, physical fitness, and exercise self-efficacy in the adult population. They were chosen because they are the largest and most recognized base of abstracts and bibliographic references in the scientific literature worldwide. This search and analysis was conducted from March to October to July 2020.

We used a pattern of argument follow-up based on the Preferred Reporting Items for Systematic Reviews and Meta-Analyses (PRISMA) protocol [30], which is recommended for the development of bibliographies, systematic reviews, and meta-analyses, where all works included in the journals in the Journal Citation Report (quartiles 1, 2, 3) and the SCImago Journal Rank (quartiles 1 and 2) were examined. 

The exhaustive review of each of the articles was managed according to author, title of article, year of publication, language, URL and/or DOI of publication, indexation of the journal, publication location, city or region of the study, number and type of sample, average age of participants, objective(s), methodology, analyses performed, measuring instruments, and techniques used.

The search terms used were: “Exercise” and “Physical Fitness” and “Self Concept” and “Self Efficacy” and “Quality of Life”, and a combination of these with the Boolean operator “AND” under the guidelines of PubMed and with the filter belonging to the PubMed database itself, limiting the age stage to “middle-aged”. The following search combinations were used: “Exercise and Physical Fitness and Self Concept and Quality of Life”, “Exercise and Physical Fitness and Self Efficacy and Quality of Life”, “Exercise and Exercise Test and Self Concept and Quality of Life”, “Exercise and Exercise Test and Self Efficacy and Quality of Life”. Criteria for the inclusion of articles were: (a) the average age of participants was within the range of 40–70 years (in the case of articles that included this data, the criterion <70 years was accepted); (b) that they were empirical articles, or (c) they were articles from bibliographical reviews. The screening of articles was done manually, and the selected papers were included in a general table (see Table 1).

## 3. Results

**Figure 1:**
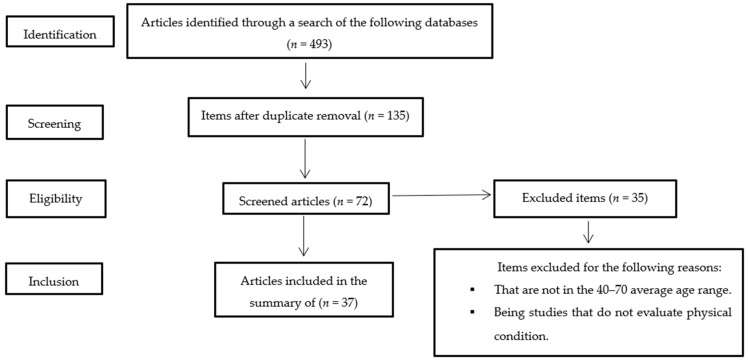
Flow diagram of article selection for the systematic review.

Figure 1 presents all the articles that were selected for this systematic review. Of the 37 articles reviewed focused on clarifying the relationship between fitness parameters and exercise self-efficacy, 32 articles were focused on populations with some type of pathology [6,31,32,33,34,35,36,37,38,39,40,41,42,43,44,45,46,47,48,49,50,51,52,53,54,55,56,57,58,59,60,61]. Five articles focused on the pathology-free middle-aged population [62,63,64,65,66]. The results obtained from the articles included in this review are shown in the Table 2 section below.

### 3.1. Results of Studies Assessing Overall Physical Fitness

Assessing physical fitness was the main objective for 12 articles, while it was secondary in 12 articles. In relation to the measurement of the instruments used, different tests have been found for the evaluation of physical fitness, of which some are general and others, specific. On 24 occasions, the test used was “The 6-Min Walk Test” (6MWT) that assesses aerobic endurance Self-perceived physical fitness was assessed on 14 occasions [6,34,37,39,40,43,47,53,56,58,59,61,65,66]. “The Foot Up and Go” test was used on six occasions [34,42,48,54,55,62]; “The Sit to Stand Test” [34,39,46,57,61,62]; “The Handgrip forced test” was used to evaluate the strength of the upper and lower body, agility in the face of possible falls, flexibility of the upper and lower body, and dynamic balance [39,42,46,57,61,63]. In five occasions “Treadmill Test” was used [6,45,60,63,64]. “The 10-Min Walk Test” (10MWT) assessing endurance was used on three occasions [40,48,50]. On two occasions the VO^2^ peak was evaluated with “The Borg Rating of Perceived Exertion Scale” [45,54]; “The Naughton Protocol” [6,47]. the “Arm Curl Test” [39,62]. In two examples these tests were used: “The test “Sit and Reach” [62,63]; “The Grip Strength Test” [53,57]; The Activities-Specific Balance Confidence Scale” (ABC Scale) [42,50]. On one occasion, the VO^2^ peak was assessed with “the Balke Protocol” [65]; “The Bicycle Ramp Protocol” [54]; “The Discontinuous Arm Crank” [38]; “The Submaximal Bicycle Ergometer” [46]; “1-Repetition Maximum Free Weight Bench Press” [38]; “The Timed Stair Climbing” [43]; “Hip Flexibility” [63]; “Functional Aerobic Impairment” (FAI) [6]; “50 Foot Flat Surface Walking Test” [43]; “Berg Balance Scale” (BBS) [48]; “The 14-item Mini Balance Evaluation Systems Test” (Mini-BESTest) [42]; “Well bench” [61].

### 3.2. Results of Studies Assessing Self-Efficacy

In 15 articles, self-efficacy was assessed as a secondary objective. On three occasions, the following instruments were used: “The Arthritis Self- Efficacy Scale” [34,43,53]; “The 16-items Cardiac Exercise Self-Efficacy” [6,54,55]; “ABC Scale” [42,50,66]. Furthermore, the following instruments were used one two occasions: “The Exercise Self-Efficacy Scale” [45,46]. On one occasion “The Self-Efficacy Questionnaire-Walking” (SEQ-W) [49] and “The chronic obstructive pulmonary disease (COPD) Self-Efficacy Scale” [52] were used. On one occasion, the following instruments were used: “The New General Self-Efficacy Scale” [63]; “8-ítems measure of beliefs capabilities” [65]; “The Physical Activity Self-Efficacy” [64]; “Self-efficacy in Leisure-Time Physical Activity” (LIVAS) [39]; “Self-Rated Abilities for Health Practices Scale” (SRAHP) [38]; “Fear of Falling Efficacy Scale” (FFES) [48]; “Self-Monitor Exercise Behavior” (SMEB) [31]; Likert Scales [62]; “General Self Efficacy Scale” (GSES) [56]; 16-Item Heart Disease Self Efficacy Scale (HDSE) [60].

### 3.3. Results of Studies Assessing Quality of Life

Quality of life has been evaluated with different instruments, 13 articles used “The SF-36 Health Questionnaire” [6,31,32,35,36,38,41,43,44,45,53,60,61]; On three occasions, the following were used: The SF-12 Health Questionnaire” [42,51,55]; “The Medical Outcomes Study 36-Item Short Form” (The MOS SF36) [44,47,52]. On two occasions “Minnesota Living with Heart Failure Questionnaire” (The MLHFQ) [54,55]; “The EuroQol Five-Dimensions Questionnaire” (EQ-5D) [46,56]; “The St. George Respiratory Questionnaire” (SGRQ-TS) [39,44]; “Chronic Respiratory Questionnaire”(CRQ) [36,49]; “The European Organization for Research and Training, Quality of Life Questionnaire—Core 30” (The EORTC QLQ-C30) [57,64]; “The World Health Organization Quality of Life questionnaire” (WHOQOL-BREF) [40,59] were evaluated.

On one occasion we used: “Self-Administered Quality of-Well-Being Scale” (The QWB-SA) [44]; “Cancer Rehabilitation Evaluation System-Short Form” (CARES-SF) [62]; “The Fibromyalgia Impact Questionnaire” (FIQ) [32]; “Stroke Impact Scale–16” (SIS-16) [50]; “The Parkinson’s Disease Questionnaire-8” (PDQ-8) [48]; “The Kidney Disease Quality of Life” (KDQOL-36) [51]; “The Quality of Well-Being Scale” (QWB) [49]; “The Asthma Quality of Life Questionnaire” [58].

## 4. Discussion

To find the relationship between physical fitness, the role of self-efficacy in physical exercise and physical exercise, and quality of life in the middle-aged population, the systematic review analyzed in detail works published on physical fitness, self-efficacy, and quality of life from 1997 to July 2020. The minimum age of the subjects was 30 years and the maximum age was 80, since there were studies whose age is between these values, even though the average age of the subjects studied was between 40 and 70 years old. A systematic search of the literature was carried out and 37 articles focusing on explaining these relationships were identified. Our results allow us to confirm that there is a relationship between the three explored constructs (physical fitness, quality of life, and self-efficacy in terms of improved health and healthy habits, although the relationship between the three variables in a related way is not entirely clear. 

The results have shown that, although there is scientific production that attends to the relationship between the three variables, in most cases the population evaluated is a population with some pathology. Only in some cases was the evaluated population free of pathologies [62,63,64,65,66] that a variation of the levels of physical fitness affects to the behavior in relation to the barriers towards the physical exercise and of the style of life of the population in consonance as they indicate authors as [50,62]. This is especially relevant since identifying the pathology-free population that regularly exercises and tries to achieve and/or maintain good levels of physical fitness that is one of the main objectives of the current study [67]. All this, together with the novelty of the subject of analysis, means that this subject of study has yet to be clarified and delimited, hence its importance. 

On a methodological level, the samples used for the studies was somewhat small: only one study [44] used a sample of 1631 participants, while the others had samples of fewer than 250 subjects. This is due mainly to the fact that these studies were interventions or programs development studies of populations with very specific characteristics; fewer descriptive studies analyze the relationships between the variables under study. This requires us to be cautious when considering the results of the reviewed studies.

The assessment, through evidence, of the capacities that support the physical fitness should be considered as a fundamental aspect in determining the functional capacity of the person. The physical fitness represents a significant influence on the quality of life associated with health, this being a key component in the quality of life [18,19,20]. In relation to the physical fitness variables studied, 30 articles assessed aerobic endurance, and 24 of these used the resistance test called The 6 Min Walk Test. Cardiorespiratory capacity is the main indicator of the subject’s state of physical fitness, with maximum oxygen consumption (VO2peak) being the physiological variable that best defines it in terms of cardiovascular capacity. It has been shown that a low level of physical fitness constitutes a major cardiovascular risk factor [67,68] and is a strong and independent factor in all causes of death [69]. In relation to strength, the following were evaluated: general muscle strength; lower body strength; maximum muscle strength of the muscles that mobilize the hand, knee, and elbow; grip strength; maximum strength; maximum grip strength; knee strength; muscle power. It should be noted that various transversal and longitudinal studies have verified that strength decreases with age [70,71], and this decrease is significant starting in the 50s for women and in the 30s or 40s for men [72,73]. It would therefore be advisable to introduce strength exercises into physical activity programs to slow down the process of loss of muscle mass.

On the other hand, given that many of the gestures of daily life require extensive articular paths, this capacity facilitates the functional independence of the person. For this reason, flexibility should be included in recommendations for physical exercise in this phase of life. Flexibility has been evaluated in a small number of studies, although flexibility of the lower and upper body was also assessed [41,61,62,63]. General mobility, walking and leg mobility, and agility have also been evaluated [34,40,42,43,48,50,54,62]. Static and dynamic equilibrium, which are affected by the progressive loss of sensory-motor function caused by increasing age, were assessed in several studies [42,43,48,53,55,66]. 

In summary, several studies in this review focused their efforts on understanding what makes a person more consistent in their active exercise behaviors. Many of these, through different types of intervention programs, have shown how increased health perception is linked to increased awareness of personal health status and associated with improved levels of physical fitness [46,50], improved behavior and enhanced adherence [31,39,46,53], and tolerance of sports behavior [6]. Therefore, knowledge of fitness levels can be an important tool in providing specific advice to the population [45]. Being aerobic capacity the most valued capacity and the 6-Min Walk Test the most used.

### 4.1. Self-Efficacy, Fitness, and Quality of Life

Empirical evidence supports the link between exercise self-efficacy and predictions of a variety of health-related behaviors [74,75]. The importance of physical inactivity for public health in the adult population underscores the importance of identifying those physical activity mediators and moderators that can be targeted for interventions to increase physical activity levels [76], being self-efficacy a powerful mediator between physical abilities and physical activity performance [66]. In this review, four articles focused on showing the relationship between physical fitness and exercise self-efficacy, three of which showed a positive relationship between both variables [33,47,55], while on one occasion no relationship was shown between the two [35]. Showing therefore greater tendency that supports the assertions of Bandura [77] that the actual performance of a skill is partially dependent on the perceived ability of the individual to undertake and persist in the achievement of that skill. For example, by limiting the barriers to physical exercise that lead to abandonment or non-participation [50]. These results are consistent with the findings of other studies in which exercise self-efficacy is postulated as a powerful indicator of measures of functional and reflex change in an individual’s physical fitness. It is also a determinant in the relationship between physical activity and various aspects of quality of life, including physical and mental health status and life satisfaction [23,66,78,79]. It is therefore desirable to understand in greater depth how to improve self-efficacy towards physical exercise [55]. 

One’s general sense of well-being—being aware of and feeling healthy and adjusted to one’s environmental conditions—seems to be an important requirement for developing self-awareness and a satisfying quality of life. Three studies in this review corroborated the relationships between the physical fitness variable and the quality of life variable [33,35,50]. Only Cameron-Tucker’s [35] study showed an absence of association between physical fitness and quality of life. These relationships are important because physical condition is a powerful marker of health and quality of life [20] and well-being [24], so it would be very interesting to learn more about these relationships, which have been little studied in the literature. For example, in subjects with chronic stroke, the increase in the number of steps correlates with increases in perceived physical function as a measure of quality of life [50]. On the other hand, if we take into account the importance of the dimensions evaluated for quality of life in middle age and in relation to the other variables analyzed, it should be noted that middle-aged women present more work-family complications and less social support as their perceived benefits of physical fitness increase [80]. It was also found that, among men, low mobility was associated with a lower quality of life in the psychological health domain. This is very important because increased dependence on others and reduced work capacity can be a major challenge for many men [81].

In the studies analyzed, no results have been found that analyze the relationships between the three variables, only the relationships between them two to two, and simply in one [33], the relationships between a measure of exercise self-efficacy and quality of life are analyzed, finding relationships between the 6MWT physical condition test is positively and significantly correlated with walking self-efficacy and with SF-36 physical Subscale but not with mental subscale. But it has not been analyzed, for example, the mediating role that self-efficacy or physical condition can have in relation to quality of life.

### 4.2. Review of Instruments and Measures

In relation to the instruments used in this review, 18 articles evaluated self-efficacy for physical exercise; these focus primarily on evaluating pre-behavioral processes such as change of behavior towards exercise [33,39,49], confidence in designated change towards exercise behavior [6,45,47,54,55], self-perceived capacity to develop sports behavior [45,51], confidence in designated change towards exercise behavior [6,54,55], social support for exercise behavior [46], and self-perceived barriers to exercise behavior [62,65]. Specifically, all of these results are related to Pender contributions, which link healthy behavior to the likelihood of engaging in it and one’s sense of self-efficacy. He proposed that self-efficacy for physical exercise has a decisive influence on health behavior, perceived barriers, and commitment to a plan of action [82]. During adulthood there is a slight decline in levels of self-efficacy and mastery, and these influence the perception that there are obstacles to achieving new goals [83]. Therefore, it is essential to improve beliefs about the effectiveness of physical exercise and to promote healthy behavior in the long term.

Quality of life was evaluated with different instruments. Eleven articles used “the SF-36 Health Survey”, a questionnaire that provides a clear understanding of what is being measured, how it is used, and the implications for future use. It includes most of the essential concepts for the evaluation of the general health status. It has also proved to be suitable for cross-cultural applications but may be too long for clinical use. In addition, its scoring method is more complicated. The Chronic Respiratory Questionnaire (CRQ), which is one of the available instruments to measure the general health-related quality of life in patients with chronic respiratory condition, and which has been translated into different languages [84]. On 3 occasions the SF-12 Health Questionnaire was evaluated. The SF-12 represents a plausible alternative to the SF-36 for measuring health status, showing only a minimal loss in measurement accuracy in comparation with SF-36 [85]. Other questionnaires analyzed in the results have been used on fewer occasions [46,54,55,56]. 

A relevant and conclusive aspect of our review is that a large variety of articles included intervention processes, the results of which focused on checking the possible effects of such interventions on the variables of physical fitness, self-efficacy, and quality of life. These results allow us to assume that, in most cases, the interventions that encourage on physical exercise programs offer benefits for physical fitness, self-efficacy, and quality of life when compared with the control groups, even throughout the follow-up time.

## 5. Conclusions

One of the main conclusions of this work is that the important role played by physical fitness and self-efficacy for physical exercise in achieving levels of well-being and quality of life in middle-aged and senior adults. Although one article [33] showed a positive relationship between the three reviewed constructs, the relationships between them are not completely clear. While there is no unanimity on the effects of these variables, it has been found that they are clear predictors of health, they benefit behavioral change, and they have a close relationship that can be mutually influenced. Since current research should try to identify variables that measure and moderate the practice of physical activity in the adult population, these data provide us with vital information that will allow us to deal with the serious problem of physical inactivity in favor of public health [76]. 

With the objective of promoting integral health, we should raise awareness that prevention should begin before disease appears [86]. However, one of the difficulties among the middle-aged population is lack of time, which undermines this link between personal cultivation and healthy habits. As for the limitations of the study, we should highlight the large age range of the samples examined—a result of the scarcity of studies dealing with this vital period. Likewise, most of the studies we examined referred to subjects with some kind of pathology. Finally, we would add that physical fitness and self-efficacy show a positive relationship, which is important in well-being at this age.

## Figures and Tables

**Table 1:** Description of the item selection process.

A total of 493 articles were identified in PubMed. 455 articles were excluded: 420 were duplicates; 35 did not meet the inclusion criteria: -Having an average age of 40 to 70 years old. -The standard deviation marked the ages between 30–80 years old. -They are within JCR (Q1, Q2, Q3) and SJR (Q1, Q2) magazines. -Any language of publication 37 articles were considered for detailed evaluation.

**Table 2:** Characteristics of the selected studies.

Participants	Variables	Instruments	Results
Collins et al., 2004 [6] Individuals with heart failure *N* = 31 Group 1: Aerobic Exercise Program Group 2: Control group Age Mean: 64 ± 10	**Physical Fitness Variables:** Peak oxygen consumption; Functional aerobic impairment. **Psychic Variables:** Exercise Self-efficacy: Confidence in designated change towards exercise behavior. Quality of Life: Self-perceived physical fitness; General health; Vitality; Mental health; Physical role; Emotional role; Social function; Body pain.	**Physical Fitness:** Functional Aerobic Impairment (FAI); Naughton Treadmill Test (TNT). **Self-Perceived Physical Fitness:** SF-36 Health Questionnaire.	Significant gains in fitness levels are shown for those who maintained exercise for 24 to 36 weeks. The exercise group has higher levels of self-perceived fitness. After the intervention, the group that maintained physical exercise showed significant improvements at 24 weeks in self-perceived physical fitness levels. Higher levels of self-efficacy for the intervention group after the program. These improvements are maintained in the subjects who maintained the exercise. No relationships between physical fitness parameters and self-efficacy and/or quality of life are found.
		**Exercise Self-Efficacy:** The 16-item Cardiac Exercise Self-Efficacy. **Quality of Life:** The Medical Outcomes 36-Items Short Form (SF-36).	
Bailey et al., 2016 [31] Pre-diabetic and type 2 diabetes participants *N* = 13 Group 1: Standard care (CON condition). Grupo 2: Self-monitoring intervention (SM condition). Age Mean: 61.14 ± 8.38	**Physical Fitness Variables** Cardiovascular fitness **Psychic Variables** Exercise Self-efficacy: Adherence to the exercise routine. Quality of Life: self-perceived physical fitness; General Health; Vitality; Mental Health; Physical Role; Emotional Role; Social Function; Body Pain.	**Physical Fitness:** The 6-Min Walk Test (6MWT).	The group 2 shows great effects due to the intervention. Levels of self-efficacy increase. Significant improvement in both behavior and exercise adherence. Quality of life increases in all groups throughout the intervention and follow-up. There is a significant increase in the 6MWT test in both groups. They do not refer to the relationships between physical fitness parameters and self-efficacy and/or quality of life.
		**Exercise Self-Efficacy:** Self-Monitor Exercise Behavior (SMEB). **Quality of Life:** The Short Form 36 Health Survey.	
Baptista et al., 2012 [32] Patients with Fibromyalgia *N* = 80 Group 1: Dance group Group 2: Control group Age Mean Group 1: 49.5 Group 2: 49.1	**Physical Fitness Variables** Functional capacity **Psychic Variables** Quality of Life: Self- perceived physical fitness; General health; Vitality; Mental health; Physical role; Emotional role; Social function; Body pain; Physical function and Severity of symptoms.	**Physical Fitness:** The 6-Min Walk Test (6MWT).	The intervention was followed by improved physical fitness and increased quality of life for the experimental group. No correlation is reported between the 6MWT test and quality of life parameters.
		**Quality of Life:** The Quality of Life Short Form 36 (SF-36); Fibromyalgia Impact Questionnaire (FIQ).	
Belza et al., 2001 [33] People with chronic obstructive pulmonary disease (COPD) *N* = 63 patients Age Mean: 65.4 ± 8.0	**Physical Fitness Variables** Functional capacity **Psychic Variables** Exercise Self-efficacy: self-perceived functional capacity. Quality of Life: Dyspnea; Fatigue; Emotional function; Mastery; Generic health status.	**Physical Fitness:** The 6-Min Walk Test (6MWT).	The 6MWT is positively and significantly correlated with walking self-efficacy (r = 0.68) and with SF-36 physical Subscale (r = 0.67) but not with mental subscale. SEQ-W is positively and significantly correlated with SF-36 physical subscale (r = 0.67).
		**Exercise Self-Efficacy:** The Self-Efficacy Questionnaire-Walking (SEQ-W). **Quality of Life:** The Chronic Respiratory Disease Questionnaire (CRQ); SF-36 Health Questionnaire.	
Bieler et al., 2017 [34] Participants with hip osteoarthritis *N* = 152 Group 1: Nordic WalkingGroup 2: strength training Group 3: Home-based exercise Age Mean Group 1: 70.0 ± 6.3 Group 2: 69.6 ± 5.4 Group 3: 69.3 ± 6.4	**Physical Fitness Variables** Functional performance: Endurance capacity; muscle strength, muscle function. **Psychic Variables** Self-perceived Physical Fitness: physical function. Exercise Self-efficacy: Self-efficacy for climbing stairs; Pain; self-perceived physical fitness and other symptoms. Quality of Life: Self-perceived physical fitness; General health; Vitality; Mental health; Physical role; Emotional role; Social function; Body pain.	**Physical Fitness:** The 30-s Chair Stand Test (30Scs); Timed Stair Climbing Test (TSC); 8-Foot Up and Go Test;15-Sencond Marching on the Spot Test; 6-Min Walk Test (6MWT). **Self-Perceived Physical Fitness:** The Arthritis Self-Efficacy Scale (ASES).	The Nordic Walking group gets the most important improvements in terms of physical fitness. Self-efficacy and quality of life also improve the most in this particular group in terms of mental health levels. The improvement in the hours spent in the most vigorous physical activity during the follow-up period is maintained. The strength training group improves functional performance and quality of life factors at 12 months, more than group 3. Quality of life improves more in group 1 and group 2 than in group 3. They do not refer to the relationships between physical fitness parameters and self-efficacy and/or quality of life.
		**Self-efficacy:** Task-Specific Self-Efficacy; The Arthritis Self-Efficacy Scale (ASES). **Quality of Life:** The Danish SF36 Health Survey.	
Cameron-Tucker et al., 2014 [35] Outpatients with chronic obstructive pulmonary disease (COPD). *N* = 84 Group 1: Chronic Disease Self-Management Program (CDSMP)+exercise. Group 2: (CDSMP)-only. Age Mean: 65.8 ± 9.35 Group 1: 64.5 ± 9.13 Group 2: 67.1 ± 9.41	**Physical Fitness Variables** Physical capacity **Psychic Variables** Exercise self-efficacy: Confidence to exercise behaviour. Quality of Life: Self-perceived physical fitness; General health; Vitality; Mental health; Physical role; Emotional role; Social function; Body pain.	**Physical Fitness:** The 6-Min Walk Test (6MWT).	There was a significant improvement in the 6MWT test for both groups. No difference in the comparison between groups. There was no change in both groups in self-efficacy. Physical fitness and the role and physical component of quality of life increased in the exercise group with no difference in treatment. They found no significant correlation between the 6MWT test and self-efficacy and quality of life. Moderate exercise and self-efficacy explained 7.9% of the variation in 6MWT in a multiple linear regression model.
		**Exercise Self-efficacy:** Exercise Self-Efficacy Scale. **Quality of Life:** The Short-Form 36 Questionnaire, version 2 (SF-36).	
Donesky-Cuenco et al., 2009 [36] People with chronic obstructive pulmonary disease (COPD) *N* = 29 Group 1: Yoga Program Group 2: Usual care Control Age Mean Group 1: 72.2 ± 6.5 Group 2: 67.7 ± 11.5	**Physical Fitness Variables:** Muscle endurance; Muscle strength; Exercise performance. **Psychic Variables:** Quality of Life: Self-physical fitness; General health; Vitality; Mental health; Physical role; Emotional role; Social function; Body pain. Dyspnea; Fatigue; Mastery, Emotional function.	**Physical Fitness:** The 6-Min Walk Test (6MWT); Symptom-Limited Test; Isokinetic muscle testing.	After 3 months of intervention with a yoga program, significant improvements are obtained in the 6MWT test for the experimental group. But they do not refer to the relationships between the parameters of physical fitness and quality of life.
		**Quality of Life:** SF-36 Health Questionnaire; The Chronic Respiratory Disease Questionnaire (CRQ).	
Feldstain et al., 2016 [37] Advanced cancer patients *N* = 80 Group 1: Quasi-experimental Average age: 64.04 ± 12.50	**Physical Fitness Variables** Maximal oxygen uptaken **Psychic Variables** Self-efficacy: Self-perceived physical fitness and general self-efficacy.	**Physical Fitness:** The 6-Min Walk Test (6MWT). **Self-perceived physical condition:** General Self-efficacy Scale.	The intervention helps to increase exercise levels and reinforce beliefs of self-efficacy. They do not study the changes that occur with 6MWT results. Self-efficacy is the only factor in the intervention that helps reduce depressive symptoms. Exercise and physical endurance are not significant in relation to depression. They do not refer to the relationships between physical fitness parameters and self-efficacy.
Froehlich-Grobe et al., 2014 [38] Wheelchair users *N* = 128 Group 1: The Staff Supported Intervention group Group 2: Self-Guide Comparison Group Age Mean Group 1: 46.0 ± 12.1 Group 2: 42.9 ± 13	**Physical Fitness Variables** Maximal strength; Aerobic capacity. **Psychic Variables** Exercise Self-efficacy: Exercise; Nutrition; Responsible health practice; Psychological well-being. Quality of Life: Self-perceived physical fitness; General health; Vitality; Mental health; Physical role; Emotional role; Social function; Body pain.	**Physical Fitness:** 1-Repetition Maximum Free Weight Bench Press; Discontinuous Arm Crank Test with SciFit Pro I ergometer.	Group 1 increased their physical exercise practice more than group 2, but there were no significant differences in the aerobic capacity or strength. Exercise Self-efficacy improved for the self-guided group. There are no changes in quality of life associated with body pain. But they do not refer to the relationships between physical fitness parameters and self-efficacy and/or quality of life.
		**Self-efficacy:** Self-Rated Abilities for Health Practices Scale (SRAHP). **Quality of Life:** SF-36 Health Questionnaire.	
Hospes et al., 2009 [39] Patients with chronic obstructive pulmonary disease (COPD) *N* = 35 Group 1: Exercise Counseling Group 2: Usual Care Age Mean Group 1: 63.1 ± 8.3 Group 2: 61.2 ± 9.1	**Physical Fitness Variables** Leg strength; Arm strength; Grip force; Cardiorespiratory endurance. **Psychic Variables** Exercise Self-efficacy: Perceived flexibility; Reaction time; Perceived general strength; Self-perceived physical condition; Smooth movements; Climbing stairs; Perceived strength in hand; Perceived speed of walking; Change in exercise behavior; Perceived balance; Perceived general activity. Quality of Life: Symptoms; Activity; Impacts. Symptoms; Functional state; Mental state.	**Physical Fitness:** The Chair-Stand-Test; The arm curl Test; The 6-Min Walk Test (6MWT); Handheld Dynanometer. **Self-Perceived Physical Fitness:** Self-efficacy in Leisure-Time Physical Activity (LIVAS)	The program carried out was effective, increasing adherence to daily physical exercise. The experimental group presented significant improvements in leg and arm strength, self-efficacy and quality of life. They do not refer to the relationships between physical fitness parameters and self-efficacy and/or quality of life.
		**Exercise Self-Efficacy:** Self-efficacy in Leisure-Time Physical Activity (LIVAS). **Quality of Life:** The St. George Respiratory Questionnaire (SGRQ-TS); The Clinical COPD Questionnaire.	
Kersten et al., 2015 [40] People with sclerosis and stroke *N* = 20 Group 1: Experimental Group 2: Control Age Mean Group 1: 57(53–70) Group 2: 54(51–67)	**Physical Fitness Variables** Aerobic capacity **Psychic Variables** Self-perceived physical fitness: Self-reported mobility. General Self-efficacy: The stable feeling of personal competence to effectively handle a wide variety of stressful situations. Symptom control; Role function; Emotional functioning and communication with physicians. Quality of Life: Mastery; Physical; Psychological; Social; Environmental	**Physical Fitness:** The 10-Min Walk Test (10MWT) **Self-perceived physical fitness:** Rivermead Mobility Index	The experimental group walks faster than the control group and these values are maintained throughout 12 months of follow-up. There are no significant changes in mobility outcomes for any of the groups. The experimental group obtains better levels of self-efficacy although the values are balanced with the control group over 12 months of follow-up. Quality of life levels are increasing in both groups. No reference is made to the relationships between physical fitness parameters and self-efficacy and/or quality of life.
		**General Self-Efficacy:** The General Self-Efficacy Scale; The Self-efficacy for Chronic Diseases Scales. **Quality of Life:** The World Health Organization Quality of Life questionnaire (WHOQOL-BREF).	
Hea-Young, 2006 [41] People with disabilities *N* = 40 Group 1: Experimental Group 2: Control Age Mean: 53.7 Group 1: 55.1 ± 13.68 Group 2: 52.29 ± 12.11	**Physical Fitness Variables:** Maximum muscle strength of the knee; Grip force; flexibility. **Psychic Variables:** Exercise self-efficacy: Performance achievements, indirect experience, verbal persuasion and physiological states. (Bandura, 1977). Locus of control; Personal control; Social desire; Ego strength; Interpersonal competence and self-esteem. Sherer and Maddux, (1982) and (1994). Quality of Life: Self-perceived physical fitness; General health; Vitality; Mental health; Physical role; Emotional role; Social function; Body pain.	**Physical Fitness:** Lafayette instrument company (United Stated of America).	After the intervention, the experimental group shows better levels of maximum muscle strength of the extensors and flexors of the knee and better levels of flexibility, in addition there are improvements in the level of self-efficacy towards exercise and in the levels of quality of life regarding the control group. They do not refer to the relationships between physical fitness parameters and self-efficacy and/or quality of life.
		**Exercise Self-efficacy:** Bandura Self-Efficacy Scale 1997; Sherer & Maddux Self-Efficacy Scale (1982) and (1994). **Quality of life:** The Short Form-36 Health Survey (version 2).	
Liao et al., 2016 [42] Chronic stroke participants *N* = 84 Group1: Low Intensity Body Vibration Group 2: Hight Intensity Body Vibration Group 3: Control Age Mean: 61.2 ± 9.2	**Physical Fitness Variables** Muscle strength; Balance; Walking endurance; Functional mobility. **Psychic Variables** Balance Self-efficacy: Confidence in the performance of specific outpatient activities. Quality of Life: Self-perceived physical fitness; Physical Role; Body Pain; General Health; Vitality; Social Function; Emotional Role; Mental Health.	**Physical Fitness:** Dynamometer; The 14-item Mini Balance Evaluation Systems Test (Mini-BESTest); The 6-Min Walk Test (6MWT); Timed Up and Go (TUG).	The results showed a significant increase between groups in the parameters of physical fitness, self-efficacy and quality of life with respect to effect size. There are no appreciable differences between group 1 and group 2 in relation to the variables evaluated. The programs are not effective in their purpose. No results are presented for the relationships between physical fitness, self-efficacy and quality of life.
		**Balance Self-efficacy:** The Activities-Specific Balance Confidence Scale (ABC Scale). **Quality of Life:** Short-Form Health Questionnaire SF-12.	
McKay et al., 2012 [43] Patients undergoing total knee arthroplasty *N* = 22 Group 1: Intervention Group 2: Control Age Mean Group 1: 63.5 ± 4.93 Group 2: 60.58 ± 8.05	**Physical Fitness Variables** Quadriceps strength; Mobility; Balance. **Psychic Variables** Self-efficacy: Pain; Self-perceived physical function; other symptoms. Quality of life: Physical function; General health; Vitality; Mental health; Physical role; Emotional role; Social function; Body pain.	**Physical Fitness:** Isometric Strength Assessment; 50-Feet Flat Surface Walking Test; Stair Ascent-Descent. **Self-Perceived Physical fitness:** The Arthritis Self-Efficacy Scale.	The intervention affects the improvement of quadriceps strength levels and significantly improves self-efficacy and quality of life for the experimental group. But they do not refer to relationships between physical fitness parameters and self-efficacy and/or quality of life.
		**Self-efficacy:** The Arthritis Self-Efficacy Scale. **Quality of Life:** The Short Form 36 (SF-36).	
Moy et al., 2009 [44] Persons with severe chronic obstructive pulmonary disease (COPD) *N* = 1621 patients. Age Mean: 66 ± 6	**Physical Fitness Variables** Exercise capacity **Psychic Variables** Quality of Life: Self-perceived physical fitness; Physical role; Body pain; General health Perceptions; Vitality; Social function; Emotional role; Mental health.	**Physical Fitness:** The 6-Min Walk Test (6MWT).	Physical fitness values are positively related to quality of life. The self-perception of being disabled is significantly associated with the quality of life.
		**Quality of life:** The Medical Outcomes Study 36-Item Short Form (The MOS SF-36); The St. George’s Respiratory Questionnaire Total Score (SGRQ-TS); Self-Administered Quality of-Well-Being Scale (QWB-SA).	
Nam et al., 2012 [45] People with type 2 diabetes *N* = 140 Group 1: Exercise Group 2: control Age Mean Group 1: 57.24 ± 6.08 Group 2: 55.53 ± 6.49	**Physical Fitness Variables** Maximum oxygen consumption; Muscle strength. **Psychic Variables** Exercise Self-efficacy: Self-perceived ability to perform arm and leg tasks before and after training. Quality of Life: Self-perceived physical fitness; General health; Vitality; Mental health; Physical role; Emotional role; Social function; Body pain.	**Physical Fitness:** Peak Oxygen Uptake (VO_2_) with Treadmill Walking Test. The Borg Rating of Perceived Exertion Scale; 1-Repetition Maximum of 7 Exercises.	Subjects who participated in the exercise group dropped out of the activity to a greater extent than in the control group; those who dropped out had lower levels of self-efficacy in lifting and less physical fitness. It does not relate physical fitness variables to self-efficacy and quality of life.
		**Exercise Self-Efficacy**: The Exercise Self-Efficacy Scale. **Quality of Life:** Short-form 36 Item Health Survey.	
Nordgren et al., 2015 [46] Rheumatoid arthritis patients *N* = 220 Group 1: Completed the program Group 2: They didn’t complete the program. Age Mean Group 1: 58 ± 9.9 Group 2: 60 ± 8.4	**Physical Fitness Variables** Maximal aerobic capacity; Lower limb function Maximum and average grip strength. **Psychic Variables** Exercise Self-Efficacy: Social support (family, friends) for exercise behavior; Expected long-term health; Beliefs to avoid fear. Quality of Life: Self-care; Pain; Discomfort; Anxiety; Depression.	**Physical Fitness:** Submaximal Bicycle Ergometer; The Timed-Stands Test; The Grippit Device.	The results showed significant changes before and after the intervention programs for the two groups. Levels of physical fitness, self-efficacy and quality of life were significantly improved at one year and greater adherence to the training program was shown, resulting in improved perception of health and self-efficacy towards exercise. No reference is made to the relationships between physical fitness parameters and self-efficacy and/or quality of life
		**Exercise Self-efficacy:** The Exercise Self-efficacy Test. **Quality of Life:** The EuroQol Five-Dimensions Questionnaire (EQ-5D).	
Oka et al., 1999 [47] Patients with heart failure *N* = 40 patients Age Mean: 56 ± 12	**Physical Fitness Variables** Functional capacity **Psychic Variables** Self-efficacy: Confidence to carry out the behavior; Average strength; Expectations of self-efficacy for each behavior.	**Physical Fitness:** VO^2^ Peak Naughton Protocol; The 6-Min Walk Test (6MWT). **Self-Perceived physical fitness:** 5-Item Physical Condition Questionnaire.	There are positive correlations between physical fitness through the 6-Min test with the walking and stair-climbing self-efficacy scales. Perceived physical fitness was associated with emotional wellbeing. No correlation was found between self-efficacy and quality of life.
	Self-Perceived physical fitness: Individual perception of various aspects of physical condition. Quality of Life: Energy; Fatigue; Wellbeing.	**Exercise Self-efficacy**: The Self- Efficacy Expectation Scales for Walking, Stair climbing and General Activities. **Quality of life:** The Medical Outcomes Study 36-Item Short Form (SF-36).	
Pilleri et al., 2015 [48] People with Parkinson disease *N* = 20 Group 1: (robot assisted gait training) Age Mean: 64.5 (45–71)	**Physical Fitness Variables** Aerobic capacity; Balance. **Psychic Variables** Self-efficacy: Fear of falling during daily activities. Quality of Life: Activities of daily life; Attention and work memory; Communication; Depression; Quality of life; Social relationship.	**Physical Fitness:** Timed Up and go Test (TUG); The 10-Min Walk Test (10-MWT); Berg Balance Scale (BBS).	After the intervention, aerobic capacity and balance improve, indicating an improvement in perceived stability. It also reflects improved levels of self-efficacy and quality of life. No relationships are expressed between the variables of physical fitness, self-efficacy and quality of life.
		**Self-Efficacy:** The Fear of Falling Efficacy Scale (FFES) **Quality of Life:** The Parkinson’s Disease Questionnaire-8 (PDQ-8).	
Ries et al., 2003 [49] Patients with chronic lung disease *N* = 172 Group 1: Experimental maintenance program Group 2: Standard care control group Age Mean: 67.1 ± 8.2	**Physical Fitness Variables** Maximum distance possible in 6 Mins. **Psychic Variables** Exercise self-efficacy: Change in behavior toward exercise (range of activity; general effort in moving things; lifting; climbing stairs; tolerating stress; tolerating anger). Quality of Life: Self-perceived physical fitness; mental function; fatigue; dyspnea; mastery. physical Functioning; body pain; role limitations due to physical health problems; role limitations due to personal or emotional problems; general mental health; social functioning; energy; fatigue; general health perceptions.	**Physical Fitness:** The 6-Min Walk Test (6MWT).	The experimental group shows improvements after the intervention in the 6MWT test; in walking self-efficacy and in quality of life levels. Follow-up over 1 and 2 years shows that the levels of resistance, self-efficacy and quality of life of the experimental group tend to be balanced with the levels of the control group. They do not refer to the relationships between physical fitness parameters and self-efficacy and/or quality of life.
		**Exercise Self-Efficacy:** The Self Efficacy Questionnaire Walking (SEQ-W). **Quality of life:** The Quality of Well-Being Scale (QWB); The Chronic Respiratory Questionnaire (CRQ); The Rand 36-ítem Health Survey.	
Sullivan et al., 2014 [50] Participants with chronic stroke *N* = 11 Group 1: Podometer-Monitored, community-based intervention Age Mean: 60.4 ± 12.1	**Physical Condition Variables** Walking endurance. **Psychic Variables** Self-efficacy: Confidence in performing specific outpatient activities. Quality of Life: Strength; hand function; activities of daily living/instrumental activities of daily living; mobility.	**Physical Condition:** The 6-Min Walk Test (6MWT); The 10-Meter Walk Test (10MWT).	The increase in the number of steps correlates with an increase in Self-perceived physical fitness and this in turn correlates with moderate changes in the 6MWT and quality of life. In addition, barriers to physical exercise are minimized. There are no significant changes in the group over the measurement time.
		**Self-Efficacy:** The Activities-Specific Balance Confidence Scale (ABC Scale). **Quality of Life:** Stroke Impact Scale-16 (SIS-16).	
Tang et al., 2017 [51] Patients with chronic kidney disease *N* = 84 Group 1: Experimental Group 2: Control Age Mean Group 1: 46.26 ± 15.61 Group 2: 43.90 ± 12.44	**Physical Fitness Variables** Endurance; Function of lower body muscle strength **Psychic Variables** Exercise Self-efficacy: Self-perceived ability to perform arm and leg tasks before and after training. Quality of Life: List of symptoms/problems; Effects of kidney disease; Burden of kidney disease; Physical component; Mental component; Physical function; General health; Vitality; Mental health; Physical role; Emotional role; Social role; Body pain.	**Physical Fitness:** The 6-Min Walk Test (6MWT); 10 Repetition of Sit to Stand Test (STS10).	No results are presented for the relationships between physical fitness, self-efficacy and quality of life. Group 1 improves their physical fitness, self-efficacy and quality of life. Improvements in 6MWT and STS10 helped to achieve the reported quality of life improvements. The exercise program is effective in improving the physical fitness and quality of life in these patients.
		**Exercise Self-Efficacy:** The Self-Efficacy for Exercise Scale (SEE). **Quality of Life:** The Kidney Disease Quality of Life (KDQOL-36); SF-12 Health Questionnaire.	
Tu et al., 1997 [52] Patients with chronic obstructive pulmonary disease (COPD) *N* = 203 Grupo 1: Subjects with unstable conditions Grupo 2: Subjects with stable conditions Group 3: Lung education subjects Age Mean: 70 years old	**Physical Fitness Variables** Functional exercise capacity. **Psychic Variables** Self-efficacy: Negative effect; intense arousal emotional; physical effort; climate/environment environment; and behavioral risk factors. Quality of life: Self-perceived physical fitness; General health; Vitality; Mental health; Physical role; Emotional role; Social function; Body pain.	**Physical Fitness:** The 6-Min Walk Test (6MWT).	Self-perceived physical fitness scale of SF-36 is more correlated with 6MWT than with emotional function. The physical fitness scale of SF-36 shows a moderate correlation between the physical fitness parameters and the physical fitness scale.
		**Self-Efficacy:** Chronic Pulmonary Disease Self-Efficacy Scale (CSES). **Quality of Life:** The Medical Outcomes Study Short Form 36 (SF-36).	
Wang et al., 2018 [53] Adult with fibromyalgia *N* = 226 Group 1: Tai chi Group 2: Aerobic exercise Age Mean Group 1: 52.1 ± 13.3 Group 2: 50.9 ± 12.5	**Physical Fitness Variables** Physical function; Muscle strength and power; Balance. **Psychic Variables** Self-efficacy: Pain; self-perceived physical fitness and other symptoms. Quality of Life: Self- perceived physical fitness; General health; Vitality; Mental health; Physical role; Emotional role; Social function; Body pain.	**Physical Fitness:** The 6-Min Walk Test (6MWT), Balance Test; The Chair Stand Test; Leg Press. **Self-Perceived Physical Fitness:** The Arthritis Self-Efficacy Scale (ASES).	No results are presented for the relationships between physical fitness, self-efficacy and quality of life. The group 1 obtains equal or better results in self-efficacy and quality of life after 24 weeks and greater adherence compared to group 2. Psychological benefits may be associated with longer exercise practice affecting mental health and physical fitness.
		**Self-efficacy:** The arthritis self-efficacy scale (ASES). **Quality of Life:** The Short Form Health Survey	
Yeh et al., 2011 [54] Patients with chronic heart failure *N* = 100 Group 1: Tai chi group Group 2: Education group Age Mean Group 1: 68.1 ± 11.9 Group 2: 66.6 ± 12.1	**Physical Fitness Variables** Aerobic capacity; Agility. **Psychic Variables** Exercise Self-efficacy: confidence in the designated change towards exercise behavior. Quality of Life: Swelling in the ankles; Difficulty climbing stairs; Fatigue; Depressive feelings; Monetary expense; Health-related treatment.	**Physical Fitness:** Bicycle Ramp Protocol (Borg scale); The 6-Min Walk Test (6MWT); Timed Up and Go.	The intervention group after the Tai chi program significantly improves the levels of quality of life and self-efficacy towards exercise in comparison with the control group. There is improvement in the 6MWT test for the intervention group although there are no relevant differences between groups. There are no relationships between physical fitness parameters with self-efficacy and/or quality of life.
		**Exercise Self-Efficacy:** The 16-Item Cardiac Exercise Self-Efficacy. **Quality of Life:** Minnesota Living with Heart Failure Questionnaire (MLHFQ).	
Yeh et al., 2016 [55] Patients with heart failure *N* = 100 Group 1: Tai chi Group 2: Education Age Mean Group 1: 69 Group 2: 66	**Physical Fitness Variables** Aerobic capacity; Agility. **Psychic Variables** Exercise Self-efficacy: Confidence in the designated change towards exercise behavior Quality of Life: self-perceived physical fitness; Physical Role; Body Pain; General Health; Vitality; Social Function; Emotional Role; Mental Health; Ankle swelling; Difficulty climbing stairs; Fatigue; Depressive feelings; Money spent; Health-related treatment	**Physical Fitness:** Bicycle Ramp Protocol; The 6-Min Walk Test (6MWT); Timed Up and Go.	There was no improvement in physical fitness. Group 1 shows significant improvements in self-efficacy over 1 year in comparison with group 2. Quality of life levels are higher in group 1 compared to group 2. The Tai chi program was effective in improving self-efficacy and quality of life with respect to the other group in this type of patient. The 6MWT test is associated with change in self-efficacy.
		**Exercise Self-Efficacy:** The 16-item Cardiac Exercise Self-Efficacy. **Quality of Life:** Minnesota Living with Heart Failure Questionnaire (MLHFQ); SF-12v2 Short Form Health Survey.	
Zanaboni et al., 2016 [56] Patients with chronic obstructive pulmonary disease (COPD) *N* = 120 Group 1: telerehabilitation Group 2: Treadmill Group 3: control Age Mean: Between 40-80 years old	**Physical Fitness Variables** Functional exercise capacity **Psychic Variables** Exercise Self-efficacy: maintenance of exercise; maintenance of self-management routines. Quality of Life: self-care; pain; discomfort; anxiety; depression.	**Physical Fitness:** 6-Min Walking Distance (6MWD).	No results are presented for the relationships between physical fitness, self-efficacy and quality of life. The physical fitness and quality of life in group 1 improves over one year compared to the other two groups. Telerehabilitation can prevent deterioration, improve physical performance, health status and quality of life.
		**Self-Efficacy:** The Generalized Self-Efficacy Scale (GSES). **Quality of Life:** The EuroQol Five-Dimensional Questionnaire (EQ-5D).	
Cheong et al., 2018 [57] Colorectal cancer patients *N* = 75 Age Mean: 58.27 ± 11.74	**Physical Fitness Variables** Physical performance Upper extremity muscle strength **Psychic Variables** Overall health status; area of functioning; area of symptoms.	**Physical Fitness:** The grip strength test; 30 s CST; the 2-min walk test (2MWT). Hand-held dynamometer.	The lower extremity strength and cardiorespiratory endurance was significantly improved. There are no relationships between physical fitness parameters with quality of life.
		**Quality of life:** European Organization for Research and Treatment of Cancer Quality of Life Questionnaire C30 (EORTC QLQ-C30).	
Coelho et al., 2017 [58]. Asthmatic women *N* = 66; Age Mean Asthma group: 45.8 ± 12.3 Control Group: 44.3 ± 11.6	**Physical Fitness Variables** Submaximal exercise capacity **Psychic Variables** Limitation of usual activities; symptoms; emotional function; environmental stimuli.	**Physical Fitness:** The 6MWT.	Daily life physical activity correlated with QoL and 6MWT. There are no relationships between physical fitness parameters with quality of life.
		**Quality of life:** The Asthma Quality of Life Questionnaire (AQLQ).	
Costa et al., 2018 [59] Schizophrenia patients *N* = 114 Age Mean: 44.25 ± 9.72	**Physical Fitness Variables** Functional exercise capacity **Psychic Variables** Physical; psychological; social relationship; environment.	**Physical Fitness** The 6MWT	QoL correlated with physical activity. Active behaviours could improve QoL. There are no relationships between physical fitness parameters with quality of life.
		**Quality of life** WHOQOL-BREF	
Moreno-Suarez et al., 2020 [60] Patients with Left Ventricular Assist Device and Patient Chronic Heart Failure *N* = 32 Age Mean LVAD Group: 59.1 ± 10.8 CHF Group: 58.3 ± 8.7	**Physical Fitness Variables** Cardiopulmonary exercise testing **Psychic Variables** Self-Efficacy: The strength of efficacy beliefs **Quality of life:** Self-perceived physical fitness; general health; vitality; mental health; physical role; emotional role; social function; body pain.	**Physical Fitness** Treadmill exercise test	Patients with LAVD reported better QoL. There are no relationships between physical fitness parameters with self-efficacy and/or quality of life.
		**Self-efficacy** 16 item Heart Disease Self-Efficacy Scale (HDSE) **Quality of life** SF-36	
Rosa et al., 2018 [61] Hemodialysis patients *N* = 52 Age Mean: 55.7 ± 14.03	**Physical Fitness Variables** Physical capacity and strength; leg and back flexibility. **Psychic Variables** Self-perceived physical fitness; general health; vitality; mental health; physical role; emotional role; social function; body pain.	**Physical Fitness** The 6MWT; Hand grip dynamometry; Sit to stand test (STS10); Wells bench.	The program increases leg lean mass and STS10 performance. There are no relationships between physical fitness parameters with self-efficacy and/or quality of life.
		**Quality of life** SF-36	
Damush et al., 2006 [62] Breast cancer survivors. Group 1: Experimental *N* = 34 patients Age Mean: 59.6 ± 6.6	**Physical Fitness Variables** Aerobic capacity, Lower body strength; Agility; flexibility; health. **Psychic Variables** Exercise for self-efficacy: Perceived barriers; Benefits and enjoyment of physical activity. Quality of Life: Depression; Fatigue; Physical functioning; Psychosocial functioning.	**Physical Fitness:** Senior Fitness Test Battery (2 Min Step Test; The 30s Chair Stand; The Arm Curl; The Chair sit and reach; Back scratch; 8ft Get Up and Go).	This program improves perceived barriers to exercise and physical fitness by improving endurance and strength levels, and quality of life. They do not refer to the relationships between physical fitness parameters and self-efficacy and/or quality of life.
		**Exercise Self-Efficacy:** The Self-Efficacy for Exercise Scale (SEE). **Quality of Life:** The Kidney Disease Quality of Life (KDQOL-36); SF-12 Health Questionnaire.	
Gregg et al., 2016 [63] Homeless male participants *N* = 18 Age Mean: 41.05 ± 11.32	**Physical Fitness Variables** Cardiorespiratory Fitness; Lower back hamstring; Hip flexibility; General muscle strength. **Psychic Variables** Self-efficacy: Dimension of the self-efficacy feature. Quality of Life: Well-being; Relationship with Others; Social Community; Vivid Involvement; Personal Development; Compliance.	**Physical Fitness:** 1-Mille Treadmill Walk Test; A Sit-and-Reach Test; A Grip Strength Test.	Self-efficacy is positively correlated with quality of life. There is no correlation between self-efficacy and quality of life with the physical fitness parameters studied.
		**Self-Efficacy:** The New General Self-Efficacy Scale (NGSE). **Quality of Life:** The Quality of Life Scale.	
Ligibel et al., 2012 [64] Cancer survivors *N* = 61 Group 1: Telephone-Based Exercise Intervention Group 2: Usual Care Control Age Mean: 50.0 ± 12.0 Group 1: 53.1 ± 10.8 Group 2: 55.5 ± 10.6	**Physical Fitness Variables** Functional exercise capacity **Psychic Variables** Exercise Self-efficacy: Precontemplation; Contemplation; Preparation; Action; Maintenance; Relaxation. Quality of Life: Global quality of life; Pain; Insomnia.	**Physical Fitness:** The 6-Min Walk Test (6MWT); Cycle Ergometer and Treadmill Based Exercise. **Exercise Self-Efficacy:** The Physical Activity Self-Efficacy Questionnaire.	The increase in the amount of weekly physical activity, the improvement in physical fitness, self-perceived physical fitness, and self-efficacy due to the intervention process for group 1. They do not refer to the relationships between physical fitness parameters and self-efficacy and/or quality of life.
		**Quality of life:** The European Organization for Research and Training, Quality of Life Questionnaire - Core 30, Version 3.0 (The EORTC QLQ-C30).	
McAuley et al., 2005 [65] Older sedentary adults *N* = 174 Grupo 1: Aerobic Activity Program (Walking or Stretching) Group 2: Toning Program Age Mean: 66.71 ± 5.35 Group 1: 67.42 ± 5.24 Group 2: 66.02 ± 11.48	**Physical Fitness Variables** Aerobic capacity **Psychic Variables** Self-perceived physical fitness: Physical fitness; Physical strength. Exercise Self-efficacy: Self-perceived physical fitness and self-efficacy to exercise; Perceptions of the ability to overcome barriers to exercise.	**Physical Fitness:** VO^2^ Peak Balke Protocol. **Self-perceived physical fitness:** The Perceived Importance Profile. Eight-Item Measure of Beliefs in Capabilities.	Self-efficacy is inversely related to positive well-being after the implementation of the program. But these do not refer to the relationships between the parameters of physical fitness and self-efficacy.
		**Exercise Self-efficacy:** Eight-Item Measure of Beliefs.	
Awad et al., 2019 [66] Community-dwelling individuals *N* = 40 Age Mean: 58.4 ± 1.6	**Physical Fitness Variables** Cardiovascular capacity **Psychic Variables** Balance confidence	**Physical Fitness:** The 6MWT; The 6MWT	The 6MWTotal and ABC score were each bivariately correlated with steps/d. Self-efficacy score was not significant independent predictor.
		**Self-efficacy**: Activities-specific Balance Confidence (ABC Scale)	
